# Anal dysplasia in adolescent and young adult men who have sex with men: a single-center retrospective and descriptive study (2010–2020)

**DOI:** 10.3389/fped.2023.1175476

**Published:** 2023-06-14

**Authors:** Jessica Addison, Ida Assefa, Elizabeth R. Woods, Susan Fitzgerald

**Affiliations:** Boston Children’s Hospital, Harvard Medical School, Boston, MA, United States

**Keywords:** adolescent, anal dysplasia screening, men who have sex with men, young adult, sexual health

## Abstract

**Objective:**

There are limited studies evaluating anal cytology results or the prevalence of anal human papiloma virus in adolescent and young adult (AYA) men who have sex with men (MSM). The purpose of this study was to review anal cytology screening results and determine whether abnormal findings resulted in completion of anoscopy in AYA MSM (13–26 years old).

**Patients and Methods:**

This was a retrospective study evaluating 84 anal Papanicolaou screening results among 36 AYA MSM patients aged 13–26 years who had an anal Papanicolaou test completed at an outpatient Adolescent/Young Adult Medicine Practice at Boston Children's Hospital, an urban, nonprofit, academic, free-standing children's hospital, from January 1, 2010, to December 31, 2020.

**Results:**

The findings of anal Papanicolaou screening included atypical squamous cells of undetermined significance (ASCUS) (37%), negative for squamous intraepithelial lesion (31%), inability to read (21.3%), and low-grade squamous intraepithelial lesion (10.8%). Most patients who had ASCUS results were referred for anoscopy (*n* = 28, 90.3%), and of those referred only 6.5% (*n* = 2) completed an anoscopy. Of those with low-grade squamous cell intraepithelial lesion results, 88.9% (*n* = 8) were referred for anoscopy, and among those who were referred, only 3.3% (*n* = 3) had completed an anoscopy.

**Conclusion:**

This study showed that there were abnormalities in cytology when anal Papanicolaou test screening was performed in this population, and the completion rates for anoscopy were low.

## Introduction

Several studies have shown that men who have sex with men (MSM) are at a high risk for anal cancer. Factors that increase the risk of anal dysplasia include exposure to the human papillomavirus (HPV), a high number of sexual partners, sexual debut at a young age, engaging in anoreceptive intercourse, and immunosuppression (e.g., living with HIV) (1). Studies have shown that adolescent and young adult (AYA) MSM have sexual debut at an average age of 15 years, have multiple sexual partners, and engage in condomless anoreceptive sex, which increases the risk of exposure to high-risk HPV and may lead to anal dysplasia (2, 3). There have been limited studies evaluating anal cytology results or the prevalence of HPV in AYA MSM because there are no national screening guidelines for this group (4). Prior reviews of anal dysplasia have classified MSM as a high-risk group, highlighted the absence of young MSM from anal HPV studies, and explored the cost-effectiveness of routine anal screening only to determine that more data are needed in the population (5). Current guidelines from organizations, such as the Centers for Disease Control and Prevention, state that cytology-based screening programs should only be performed if referrals to high-resolution anoscopy and biopsy are available (6).

The New York State Department of Health AIDS Institute screening guidelines for individuals living with HIV <35 years old recommend evaluating for signs or symptoms suggestive of anal dysplasia (7). The HIV Medicine Association of the Infections Disease Society does not have age parameters but recommends anal Papanicolaou test screening in individuals with a history of receptive anal intercourse only if there is access to an appropriate referral for follow-up (7). Other organizations recommend a digital anal rectal examination every 1–3 years as part of screening practices (8). There are guidelines for cervical cancer screening in women starting at age 21, as recommended by the Academy College of Obstetrics and Gynecology, even if they have never been sexually active, or at age 25 based on The American Cancer Association (9, 10).

Although anal cancer is rare before the age of 26 years in AYA MSM, more research is needed in this population to accurately depict the incidence and prevalence of anal dysplasia (11). To provide more accurate data to inform anal cancer screening recommendations in AYA MSM, this study aimed to review the anal cytology results in this population and provider-screening practices.

## Methods

Included in this study were all AYA MSM patients aged 13–26 years old who had an anal Papanicolaou test obtained at an Adolescent/Young Adult Practice affiliated with an urban, nonprofit, academic, free-standing academic children's hospital in the Northeast US (Boston Children's Hospital) from January 1, 2010, to December 31, 2020. Patients were identified using Netezza® Structured Query Language (SQL), an administrative searchable database. This database also includes patient demographics and clinical documents from the hospital's electronic medical records (EMR). The anal Papanicolaou test results were assessed using the institution's electronic medical records. This study was approved by the institutional review board of our hospital.

A chart review of MSM AYA patients who met the inclusion criteria identified through the administrative database was performed to document anal cytology results and determine whether an anoscopy referral was recommended and performed based on abnormal cytology results. Patients were excluded if they did not identify as MSM, have never engaged in anal sex, or had an anal Papanicolaou test obtained for reasons outside of sexual health screening. The principal investigator conducted a chart review focusing on the outpatient EMR Cerner PowerChart® (Kansas City, MO, United States) (12). For internal consistency, 20 charts were randomly selected and reviewed by a second reviewer and the differences were reconciled. An internal database (Hound Dog) was used to determine anoscopy recommendations by providers and whether this procedure was completed by using “anoscopy,” “anal Pap,” and “anal cytology” as search terms within an individual's EMR. The same database was used to determine if the patient identified as MSM by using the search terms “MSM” and “anal sex” in addition to a chart review of their sexual history. Microsoft® Excel (Version 16.72, for Mac) was used for data compilation, and calculation of mean age, standard deviation, years between repeat anal Papanicolaou tests, and percentages.

The demographic covariates that were extracted included the age at which the anal Papanicolaou test was obtained and race/ethnicity. Self-identified races included Black/African American, White, Another, and Unknown. Ethnicity was reported as Hispanic/Latino or non-Hispanic. Additional variables evaluated included patient insurance (private, public), history of sexually transmitted infections (STIs), type of test (urine/rectal/pharyngeal chlamydia, gonorrhea, trichomonas, and syphilis), and the number of HPV vaccines received. HPV vaccination completion was determined based on the 2011 and 2016 Advisory Committee on Immunization Practices guidelines, which stated: (1) vaccination with HPV4 is recommended for males aged 13–21 years who have not been vaccinated previously or who have not completed the three-dose series, and males aged 22 through 26 years may be vaccinated; and (2) for men who have sex with men, routine HPV vaccination is recommended for all males aged 11–26, and vaccination through age 26 years for those who were not adequately vaccinated previously (13, 14).

Anal cytology tests were performed at Associated Regional and University Pathologists, Inc. (ARUP) Laboratories (Salt Lake City, UT, United States). Cytology results were classified according to Bethesda System terminology as negative for intraepithelial neoplasia, atypical squamous cells of undetermined significance (ASCUS), low-grade squamous cell intraepithelial lesion (LSIL), and high-grade squamous cell intraepithelial lesion (HSIL) (15).

## Results

A total of 37 patients initially identified as male and had an anal Papanicolaou test obtained were included. One patient was excluded after chart review as it was determined that they did not identify as MSM and anal Papanicolaou test was performed for reasons other than sexual health screening. A total of 84 clinic encounters where anal Papanicolaou screening was performed, involving 36 patients meeting the inclusion criteria, were included in the analysis. Most of the study population was 22–24 years old (52.8%), with a mean age of 21.5 years (SD = 3.4), identified as Black/African American (44.5%), and 72.2% as non-Hispanic/Latino. Of the included patients, 75.0% completed the HPV vaccination series (Table 1). Half of the patients were living with HIV during the study period (Table 1). Table 1 outlines the history of STIs and shows that 66.7% of the patients had a positive STI history prior to their first anal Papanicolaou screening. The most prevalent STI was *Neisseria gonorrhoeae* detected in urine (14 cases), followed by rectal *Chlamydia trachomatis* (13 cases) (Table 1).

**Table 1:** Descriptive statistics of adolescent and young adult men who have sex with men, *N* = 36 (84 encounters among 36 patients).

	*N* (%)
Race	
Black/African American	16 (44.5)
White	7 (19.4)
Another	7 (19.4)
Declined to Answer/Unknown	6 (16.7)
Ethnicity	
Hispanic/Latino	10 (27.8)
Non-Hispanic	26 (72.2)
History of STI	
Yes	24 (66.7)
Number of positive STI tests	Total number, *n* = 48 (%)
Urine GC^a^	14 (29.2)
Urine CT^a^	11 (22.9)
Rectal GC^a^	6 (12.5)
Rectal CT^a^	13 (27.0)
Pharyngeal GC^a^	3 (6.25)
Pharyngeal CT^a^	1 (2.10)
Positive RPR^a^	12 (33.3)
Living with HIV	
Yes	18 (50.0)
Age when anal Papanicolaou test was obtained	
≤18	3 (8.33)
19–21	12 (33.3)
22–24	19 (52.8)
≥25	2 (5.57)
Received three HPV doses before anal Papanicolaou screening	
Yes	27 (75.0)

STI, sexually transmitted infections; HPV, human papiloma virus; GC, gonorrhea; CT, chlamydia; RPR, rapid plasma reagin; positive RPR = syphilis.

^a^ Number of cases.

**Figure 1:**
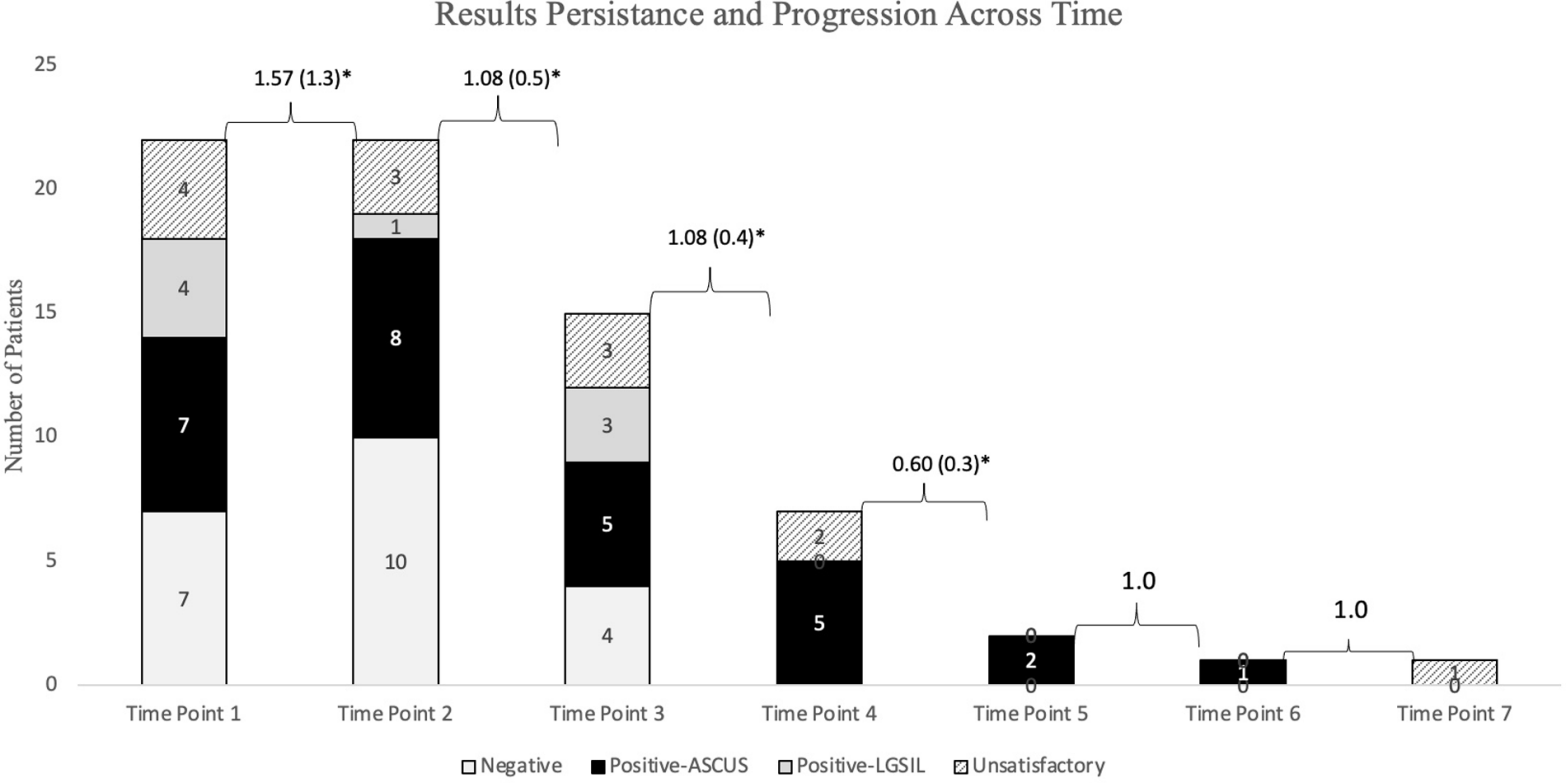
Progression and persistence of anal Papanicolaou test results in adolescent and young adult men who have sex with men patients who had repeat screening (*n* = 70 encounters with 22 patients). *Mean number of years between time points (SD).

**Figure 2:**
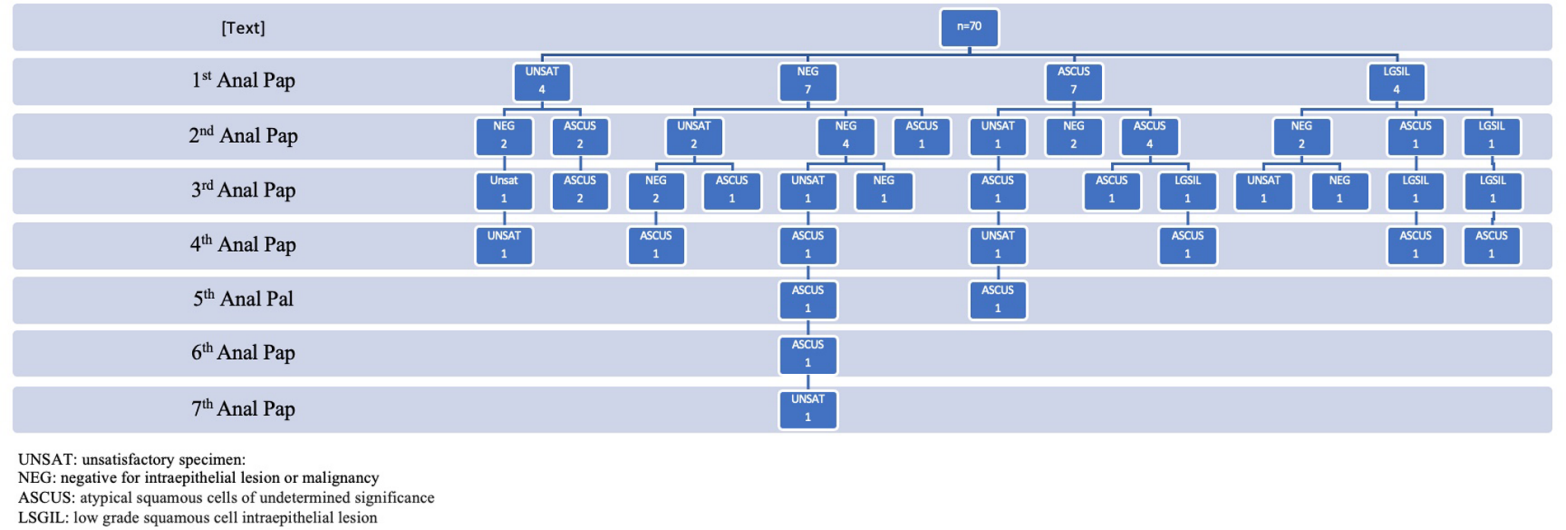
Progression and persistence of anal Papanicolaou test results in adolescent and young adult men who have sex with men patients who had repeat screening (*n* = 70 encounters for 22 patients).

The most prevalent finding on anal Papanicolaou screening was ASCUS (36.9%), followed by negative for squamous intraepithelial lesions (31%), and low-grade squamous cell intraepithelial lesion (LGSIL) (10.7%) (Table 2). Approximately 20% of screening tests returned unsatisfactory. Reasons for unsatisfactory results include contaminated sample (fecal material, bacteria), inflammation, or the specimen did not meet minimum criteria for cellularity and so could not be evaluated. Of these 36 patients, 22 (61.1%) underwent repeat anal Papanicolaou screening (during a total of 70 follow-up encounters). The time between repeat anal screening performed by providers ranged from 6 months to 2 years (mean = 1.29 years, SD = 0.9). None of the patients underwent a documented digital anal rectal examination. The majority of ASCUS cytology results remained ASCUS on the repeat anal Papanicolaou test within 2 years (Figure 1). There was only one case that progressed from negative to LGSIL within 1 year, and one case in which LGSIL cleared to a negative result within 1 year (Figure 2). Four patients regressed from LGSIL to ASCUS and three regressed from ASCUS to negative (Figure 2). Figure 2 highlights cytology progression over time and shows that ASCUS was persistent across the six time points when repeat screening occurred.

**Table 2:** Results of anal Papanicolaou screening in adolescent and young adult men who have sex with men, *N* = 84.

Anal cytology results	*N* (%)	Anoscopy recommended	Anoscopy completed
Unsatisfactory specimen	17 (20.2)	—	—
Negative for squamous intraepithelial lesion	26 (31.0)	—	—
Atypical squamous cells of undetermined significance	31 (36.9)	28/31 (90.3)	2/31 (6.45)
Low-grade squamous intraepithelial lesion	9 (10.7)	8/9 (88.9)	3/9 (33.3)
Extensive hyperkeratosis and scant nucleated squamous cells	1 (1.20)	—	—

The majority of patients who had ASCUS results were referred outside of the hospital for anoscopy (*n* = 28, 90.3%), and of those referred, only 6.45% (*n* = 2) had an anoscopy documented (Table 2). Of those with LGSIL results, 88.9% (*n* = 8) were referred for anoscopy of which 33.3% (*n* = 3) has an anoscopy documented as completed (Table 2). The reasons why patients did not undergo repeat anal Papanicolaou screening (38.3%, *n* = 14) based on chart review were as follows: (1) declined repeat screening and (2) lost to follow-up.

## Discussion

This study showed that AYA MSM often have abnormal anal Papanicolaou cytology. Many patients with ASCUS continued to have this abnormality persist for several years, which is a risk factor for progression. For the majority of patients in this study, LGSIL and ASCUS cytology self-resolved. Most studies analyzing anal dysplasia cytology are in individuals >35 years of age, where there are guidelines on how to manage abnormal anal Papanicolaou results. Findings from this study highlight the need for more long-term studies in this population to determine if a history of anal dysplasia increases the risk of anal cancer and high-risk dysplasia in the future. One study showed that in 10% of patients considered high risk (e.g., living with HIV), ASCUS on anal cytology preceded high-grade squamous cell intraepithelial lesion (HGSIL) on subsequent biopsy, highlighting that ASCUS should not be dismissed as insignificant and should be monitored (16). One meta-analysis showed the analyses of screening for anal cancer to be cost-effective in both MSM not living with HIV (testing every 3 years) and MSM living with HIV (annual testing) (17, 18). This finding was based on data from adult MSM, highlighting the paucity of data in younger populations, and suggesting that more studies are needed to include this age group in future studies. Finally, most patients in this study identified as Black/African American, which also contributes to the current literature. A meta-analysis conducted around the time of this study highlighted the lack of black/African American MSM in studies pertaining to anal cytology screening. Of the 25 studies analyzed, only two were stratified by race and showed that Black MSM had elevated rates of abnormal anal cytology, highlighting the need for additional data about these populations (19).

The Adolescent/Young Adult Practice in this study had an outside Community Health Center with expertise in LGBTQ+ care or a neighboring large academic adult hospital, both with the ability to perform an anoscopy, which is recommended in the current guidelines. Despite this fact, the success rate of having an anoscopy documented as complete at the AYA care site was very low (<5%). Common barriers to obtaining an anoscopy were still present in this study, even though there were appropriate resources. These barriers included having to go to a different institution to have the anoscopy performed, and providers not receiving results from the referral centers. Other challenges encountered were (1) transportation barriers, (2) financial concerns, (3) patients feeling uncomfortable interacting with new care teams, and (4) fear of the procedure itself. In this study, the provider recommendations for anal Papanicolaou screening were not uniform. Some recommended repeating anal Papanicolaou at 6 months to a year if the results returned as ASCUS. Other providers repeated anal Papanicolaou at 1 year if ASCUS or higher was indicated, while some referred patients directly to anoscopy when cytology indicated ASUS or higher. There were also instances where the results returned as an insufficient sample that highlights provider's lack of knowledge about performing the anal Papanicolaou procedure.

Limitations include a small sample size and the fact that the study was performed at a single large academic institution, which affected the generalizability of the results. This study demonstrated persistence in the AYA MSM population, which could lead to disease progression over time with long-term follow-up. Information could only be obtained from one institution's EMR, and the visit information may not have been transmitted back to the referral site. This may have underestimated the number of patients who completed anoscopies at other institutions and the number of subsequent abnormal test results. Long-term follow-up may not have been available for patients who were transitioning to adult care during the study period, which may have also underestimated the progression of dysplasia over time. More studies with long-term follow-up are needed in the AYA MSM adolescent population to contribute to the development of appropriate guidelines for screening anal dysplasia.

## Conclusion

This study showed that in this population of AYA MSM, there were often abnormalities in cytology when anal Papanicolaou screening was performed. In addition, there was a high rate of ASCUS with the persistence of pathology over time with multiple tests, which may be a risk for progression. Although this clinical practice had access to medical centers that performed anoscopies, the documentation of the completion of this procedure was low in this population. The findings also highlight the differences in screening practices among providers, given the lack of guidelines in this population. Follow-up of anal Papanicolaou test outcomes over time in MSM populations is critical, and tracking of results will help shape future anal dysplasia screening recommendations and the development of necessary guidelines for clinical care.

## Data Availability

The raw data supporting the conclusions of this article will be made available by the authors, without undue reservation.
